# Investigating activity of masticatory muscles in patients with hypermobile temporomandibular joints by using EMG

**DOI:** 10.4317/jced.52125

**Published:** 2015-04-01

**Authors:** Amin Davoudi, Abbas Haghighat, Oleg Rybalov, Elham Shadmehr, Amin Hatami

**Affiliations:** 1 Dentistry Student, Dental Students Research Center, School of Dentistry, Isfahan University of Medical Sciences, Isfahan, Iran; 2 Assistant Professor of Oral and Maxillofacial Surgery Dental Implants Research Center, Department of Oral and Maxillofacial Surgery, School of Dentistry, Isfahan University of Medical Sciences, Isfahan, Iran; 3 MD Professor of Oral and Maxillofacial Surgery, Department of Surgical Dentistry and Maxillofacial Surgery in plastic and reconstructive surgery of head and neck, Poltava, Ukraine; 4 Assistant Professor, Torabinejad Research Center and Departments of Endodontics, School of Dentistry, Isfahan University of Medical Sciences, Isfahan, Iran; 5 Dentist, Private practice, Isfahan, Iran

## Abstract

**Background:**

Temporomandibular joint hypermobility (TMJH) can manifest higher range of motions in mandible. The aim of this study was to investigate and compare the activity of masticatory muscle of TMJs in healthy individuals and patients with mild, moderate and severe TMJH.

**Material and Methods:**

In this clinical study, 69 patients (between the ages of 22 to 42) with manifestation of TMJH were included. The patients were divided into three groups based on their maximum mouth opening (MMO): (light) with MMO of 50-55 mm; (moderate) with MMO between 55 to 65 mm; and (severe) with MMO >65 mm. Also, 20 healthy people with profiled tomography in the last 6 months were invited as control group (healthy) with normal MMO (<50 mm). All the groups subjected to electromyogram (EMG) in 2 steps: maximal voluntary clenching (MVC) of the jaws; and during chewing of bread by using one side of the jaws voluntary. The collected data were analyzed by Student T-test and Chi-Square tests using SPSS software version 15 at significant level of 0.05.

**Results:**

Both TMJs of light, moderate and severe groups showed significant differences in frequency, time of activity and rest in comparison with healthy group during chewing and MVC (all p values < 0.01).

**Conclusions:**

Masticatory muscles activity reduced in relation with the severity of TMJH and higher excessive mouth opening.

** Key words:**Electromyography, joint hypermobility, mouth opening, tempormandibular joint.

## Introduction

Internal derangements of tempromandibular joint (TMJ) interfere with movements of the mandible smoothly, which might be due to disc displacements and tempromandibular joint hypermobility (TMJH) (1). TMJ’s range of motion might be affected by several factors: biochemical changes of collagens and elastin, loss of resistance to traction, weakness or laxity of the capsule, joint mobility and generalized joint hypermobility (GJH) (a hereditary disorder which is determined as hypermobility in multiple joints (2,3).

Winocur E *et al.* surveyed the prevalence of GJH and TMJH among adolescent girls. They stated that the prevalence of GJH and TMJH were 43% and 27.3% (3).

In another study, Oral K *et al.* found that both local and general joint hypermobility were more associated with TMJ disorders, also the risk of TMJ dysfunction would be greater if the disorders were occurred simultaneously (4).

Clinicians often diagnose hypermobility by trying some clinical exams but rarely end in precise diagnosis (5) and TMJH patients mostly complain about difficulty of mastication (2).

Electromyography (EMG) has been introduced as a tool which is employed in the diagnosis of TMJ disorders and analysis of muscle performance, physiologically (6).

Lower signals in EMG appear at rest and greater ones emerge under isometric contraction. Hence, it can be anticipate that when a muscle is suffered by some dysfunctions, the rest increases and activity signal decreases during isometric contraction (7).

Luder G *et al.* compared the muscle activity in women with normal and hypermobile joints by using EMG. They stated that lower activity was represented by EMG for the investigated muscles (quadriceps and hamstrings) in hypermobile women (8).

Due the fact that previous studies were mostly focused on administering EMG for diagnosing GJH (8) or TMJ disorders (6,7,9) not specifically TMJH, the aim of present study was to investigate the activity of masticatory muscles in patients with manifestation of TMJH.

## Material and Methods

Ethics: Present article is based on thesis with ID number of UDK: 616.724-08.089.23; the survey was executed in medical and surgical department of Poltava Dental Clinic and Maxillofacial department of POKB, Ukraine. The study protocol was approved by the ethical committee of Ukrainian Medical Stomatological Academy, Poltava. Also, a medical consent was filled by each contributors and all procedures were required for treatment plans.

This observational/case-control clinical study was conducted on 89 individuals between the ages of 22 to 42, in which 69 patients had manifestation of TMJH. Medical history and chief complaints were recorded from each patient.

The exclusion criteria were: suffering from severe systematic diseases like rheumatoid arteritis, history of maxillofacial trauma, having dentures, consumption of NSAID medications before the test, and non-cooperative patients.

For recording maximum mouth opening (MMO), the subject were asked to open their mouth as wide as possible, then a disposable ruler was used for measuring MMO. The references were the incisal edge of the upper central incisors to the incisal edge of the lower central incisors at the midline. Also, the overbite was measured and added to previous records for each individual. The measuring procedure was prepared three times for each patient and the mean of them was considered.

Then patients were divided into following three groups based on their MMO:

(Light): 25 patients with MMO of 50-55 mm.

(Moderate): 18 patients with MMO between 55 to 65 mm.

(Severe): 26 patients with MMO > 65 mm.

(Healthy): 20 individuals, with profiled tomography in the last 6 months, who were invited as control group with normal MMO (< 50 mm) (10).

EMG readings and analyses were performed according to the previous studies (7,9) by using four channels EMG with analog-digital conversion board of 16-bit, sampling frequency of 2 KHz, Butterworth filter with high-pass cut-off frequency of 10 Hz and low-pass of 1000 Hz (Neuro-EMG-Micro, Neurosoft, Ivanovo, Russia). Before collecting the EMG signal, the skin impedance was reduced by cleaning with isopropyl alcohol swab 70°. The electrodes were positioned on parallel fiber bundles of the examined muscles (right and left temporal, right and left masseter) and one reference electrode was positioned to approximately 10 mm above the glabella, according to SENIAM (Surface EMG for Non-Invasive Assessment of Muscles) (11). The Frankfurt plane was adjusted parallel to the ground and the subjects were asked to feel comfortable, with positioning their arms by their sides, looking straight ahead with no head or body movements during the test. The EMG examination was performed in 2 steps: maximal voluntary clenching (MVC) of the jaws (which has been considered as a useful approximation) (12); and during chewing by using one side of the jaws voluntary.

For evaluating MVC, the subjects were instructed to clench as hard as possible.

The TMJ, which represented more irregular patterns with lesser levels of amplitude in its exclusive EMG, was assumed as “unstable side” and the other TMJ was named “symmetric side”.

The MVC was recorded for five seconds and three times with a three-minute interval between readings.

After 5 minutes rest, standard piece of soft rye bread (which was baked one day before in size of 1×1×1 inch) was given to each individual for recording EMG during chewing. The individuals were asked to lightly and unremittingly bite with one side in the meantime of hearing beats of a metronome which was calibrated to 60 beats per minute.

Each spindle in EMG has an initial phase, the phase of optimal activity and phase of decline that goes into a period of relative rest. So, following parameters were evaluated in EMGs.

Amplitudes (mV), which was the sum of the highest and lowest signals of muscle activity and it was achieved by calculating the average sums amplitudes for each TMJ. Also, rest time (ms) and activity time (ms) of muscle were considered as periods in which the TMJ muscle were at rest or contraction.

**Figure 1:**
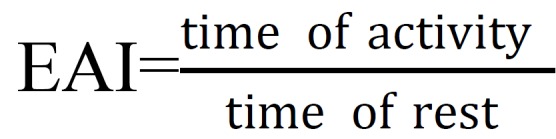
Efficacy of activity index (EAI).

Efficacy of activity index (EAI) which was obtained from the below formula: (Fig. 1).

Finally the recorded data during MVC and chewing were compared with healthy individuals by Chi-square and student T-test tests using SPSS software version 15 at significant level of 0.05.

## Results

The Chi-square test revealed that the largest number of patients with TMJH was at age of 32 to 42 years old (70.99%), while 29.01% were at the age from 22 to 31 years old. Also, the number of women was three times more (78.2%) than men (21.8%) which was significant (*p* value< 0.05).

The student T-test of EMG analysis during chewing ( Table 1) revealed that time of activity had significant difference between working and balancing TMJs in light group (*p* value = 0.01). The same result was observed in moderate group (*p* value = 0.01). However, the analysis supported significant differences between working and balancing TMJs in sever group in frequency, time of activity and rest (all *p* values <0.01). The highest and lowest amplitudes (1065.56 ± 25.72 and -830.10 ± 40.40) were observed in healthy group.

**Table 1:**
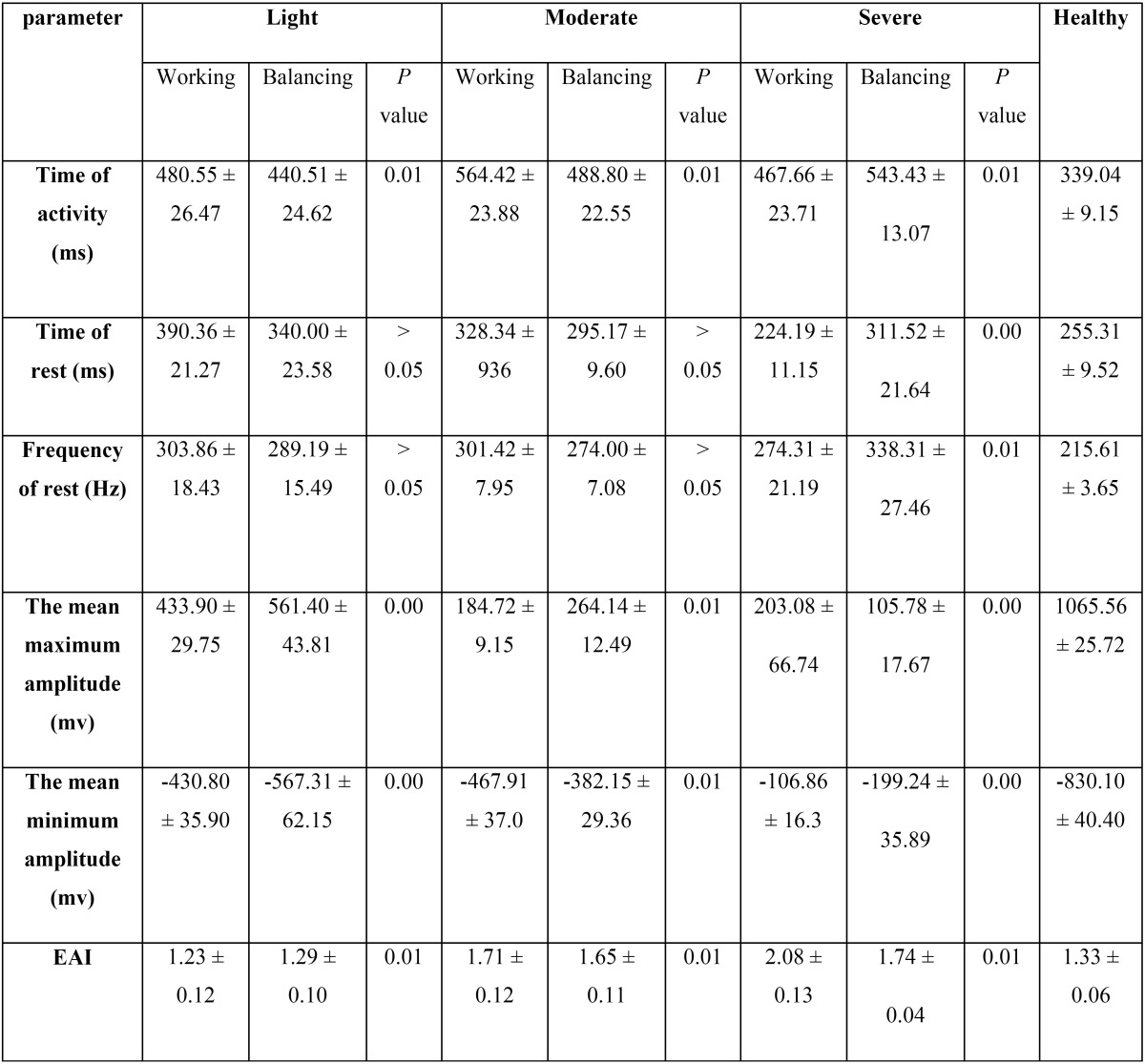
The EMG analysis of all the groups during chewing a soft ray bread.

Also, the EAI was the highest in working TMJ of sever group (2.08 ± 0.13).

 Table 2 demonstrates the EMG analysis of all the groups during MVC. Based on the results, Frequency of activity was different between unstable and symmetric TMJs significantly in light group (*p* value = 0.02). The same results were found in moderate and sever groups (all *p* values < 0.01). The highest and lowest amplitudes (968.50 ± 44.30 and -819.80 ± 31.20) were recognized in healthy group.

**Table 2:**
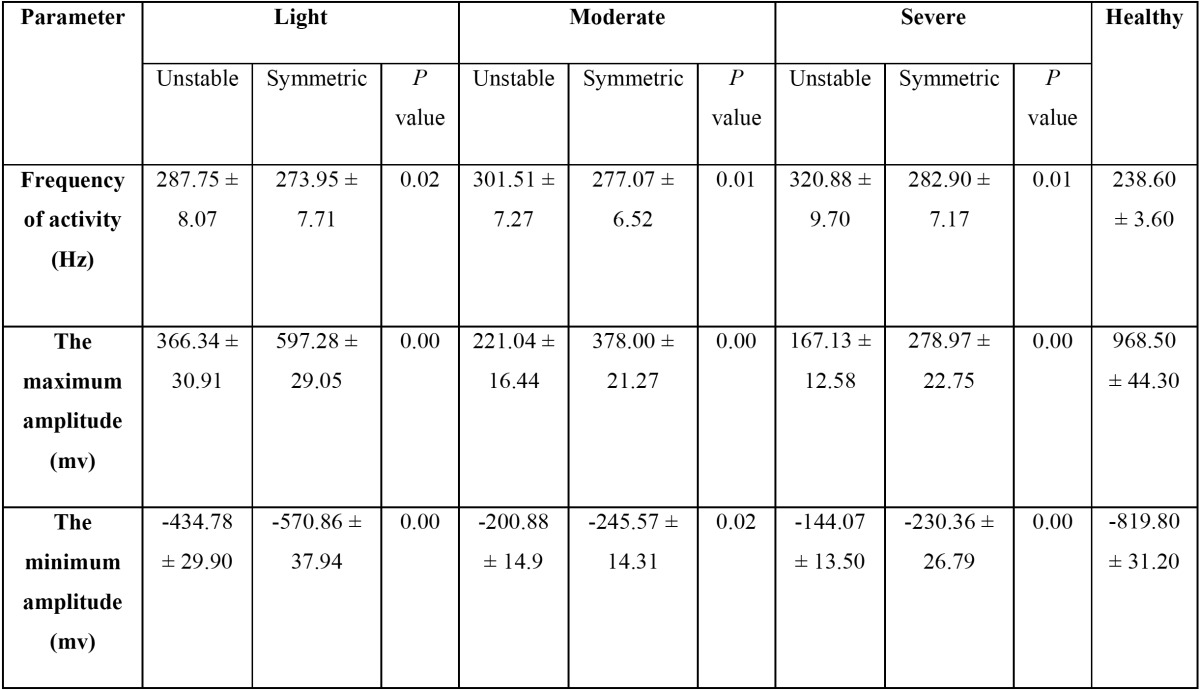
The EMG analysis of all the groups during MVC.

Due to the fact that statistical analysis of EMG did not show any significant differences during chewing and MCV between both TMJs in healthy individuals (*P* values >0.05), the average values of each parameter-which were obtained from both TMJs-were considered in healthy group. These values supposed as base lines for comparing TMJH groups with healthy condition. So,  Table 3 represents the comparisons of measured parameters between patients with TMJH (light, moderate and sever) and healthy individuals during chewing and MCV.

**Table 3:**
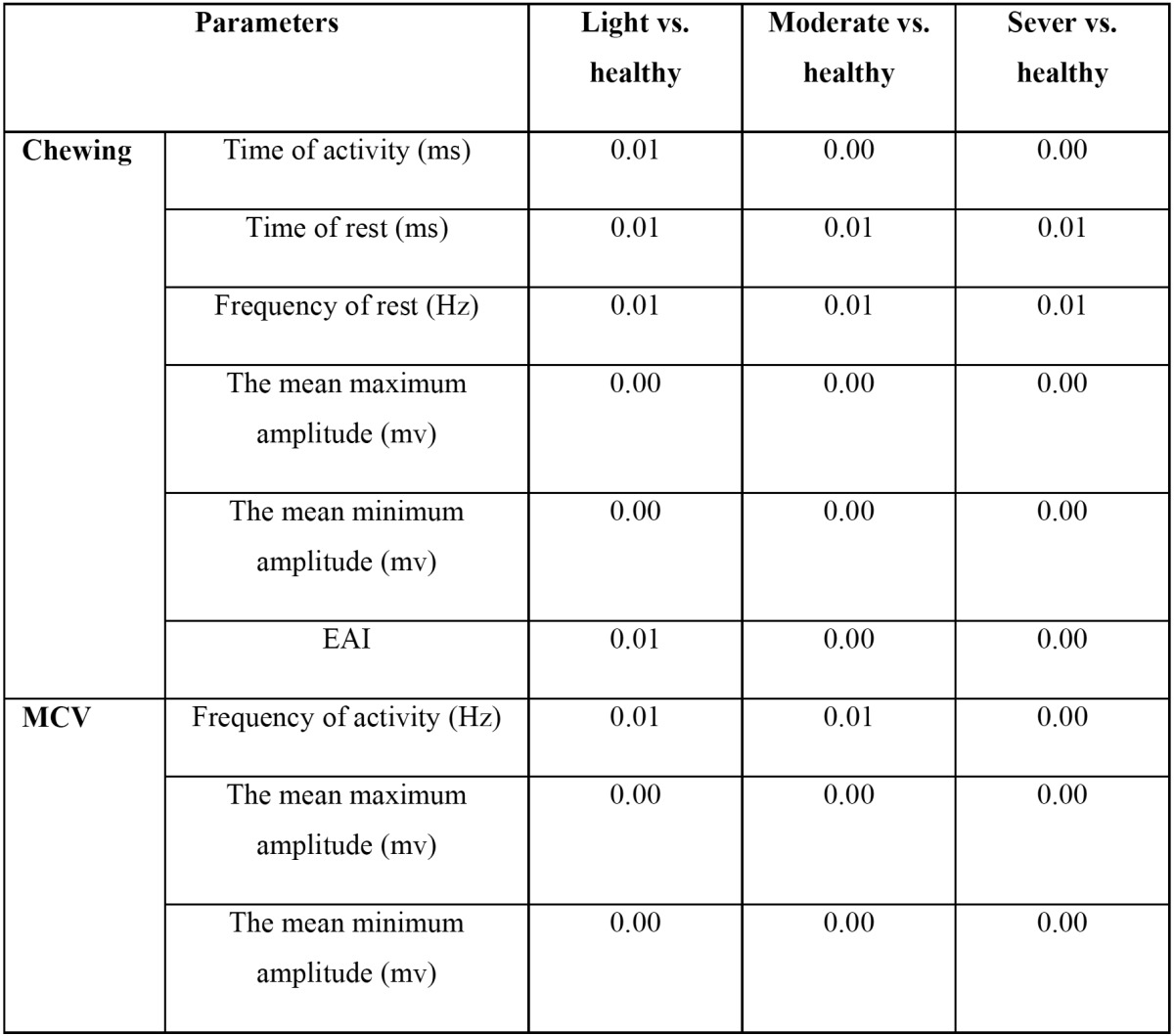
comparison of EMG parameters of TMJH groups with healthy individuals during voluntary chewing and MCV.

Both working and balancing TMJs of TMJH groups showed significant differences in frequency, time of activity and rest with healthy group during chewing (all *p* values < 0.01).

Also, both unstable and symmetric TMJs of TMJH groups demonstrated significant differences with healthy group in frequency of activity, the mean maximum and minimum amplitudes (all *p* values < 0.01) while MVC ( Table 3).

**Figure 2:**
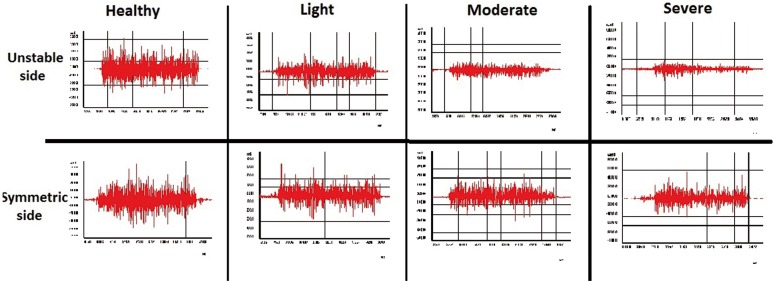
The EMG graphs of all the groups during MVC.

**Figure 3:**
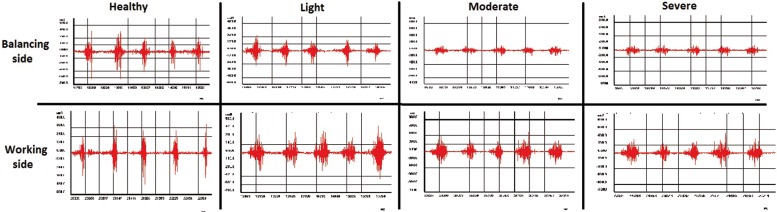
The EMG graphs of all the groups during chewing soft ray bread.

EMG research of masticatory muscles in healthy group showed minor asymmetry of bioelectrical activity in the period of MVC (Fig. 2) and during voluntary chewing (Fig. 3). Zones of activity were followed by periods of rest during chewing in healthy group. Also, amplitudes of muscle activity on working and balancing sides were visually almost identical. In light, moderate and severe groups, EMG showed asymmetry in frequency of activity, maximum and minimum amplitudes during MVC and chewing between both sides. So, fall of bioelectrical activity of muscle fibers were recognized not only in the unstable TMJ, but also from symmetric side during MVC.

## Discussion

Evaluating the EMG of masticatory muscles is currently a part of patient assessment in dental treatments (6,13), which provides quantitative data based on the function of muscles with imposing minimal discomforts.

EMG may not consider as a useful tool for diagnosing TMJ disorders (14) and its reliability and validity might hinder clinical validity if well-standardized methods were not used (6).

Based on the present results, TMJH was more common in women (74.2%) which is in accordance with some other studies (15,16).

In the current study, the quantitative EMG characteristics of masticatory muscles were differ from those healthy control groups without TMJ alterations during MVC and chewing. The highest amplitude, which reflects the significant activity and efficacy of muscle fibers, was obvious in healthy group. Recurrent sensation of pain in hypermobile joint may reduce the activation level of the muscle during daily life activities (17). Besides that, undetectable effusion may cause important inhibition of activation (18).

Increase in the periods of rest and activity of muscle fibers was obvious in both the working and balancing TMJs during chewing, which reflects an increase in EAI (especially in severe group). The higher values of EAI indicates that the muscles are obliged to contract in longer periods of time to compensate the lower biting force (amplitude) for maintaining the efficacy of chewing. This fact has been observed by some studies too (7,9,19,20).

Although there is not enough dedicated studies to TMJH, previous investigations found that the masticatory muscles of dysfunctional TMJ were less efficient and easily fatigued when they were compared to healthy subjects (19,20). So, reduced electric potentials (amplitudes) would be present in EMG graph during contraction of muscles (20-22). Also, the masticatory efficiency would be lessened and the maximum bite force reduces significantly (23).

Kimoto H *et al.* revealed that muscle conditions and adoptability might be influenced by variety of aetiological factors such as: occlusal discrepancies and isometric contractions, retention of fluids in the muscle body, blood supply and metabolic products. They suggested that improvement in the condition of the masticatory muscles leads to enhanced chewing capacity (24). Also, physiological muscle alterations have been detected in patients with TMJ disorders (9). A positive correlation has been established between hypermobile TMJ and MMO (3). The results of present study confirmed these mentioned facts, indirectly. In current study, severe group had lower activity of masticatory muscles; moreover, laxity of ligaments and muscles might be a reason of increasing distance in TMJ and positioning of condyle mostly in front or along to the articular eminence (2) and alteration in masticatory muscles. So lower chewing capacity, especially in sever group, was apparent in EMG graphs (Fig. 3) due to muscle alterations in present study.

## Conclusions

Although hypermobility is relatively common in the general population, but reports about musculoskeletal complaints are infrequent. As most symptoms are mild and self-limiting so patients may not search for medical attention (25). Based on the limitation of this study (like: medium sample size, not including other masticatory muscles, not evaluating TMJ radiographs and etc.), it can be concluded that activity of masticatory muscles was reduced in relation with the severity of TMJH; and chewing efficacy was lower in severe group in comparison with healthy people in both working and balancing sides.
